# Supplementary material for: Evidence of a one-dimensional thermodynamic phase diagram for simple glass-formers
Source: Nat Commun. 2018 Feb 6;9:518. doi: 10.1038/s41467-017-02324-3 (PMC5802781; doi:10.1038/s41467-017-02324-3)
Supplement: Supplementary file 1 — Supplementary Information [file 41467_2017_2324_MOESM1_ESM.pdf]

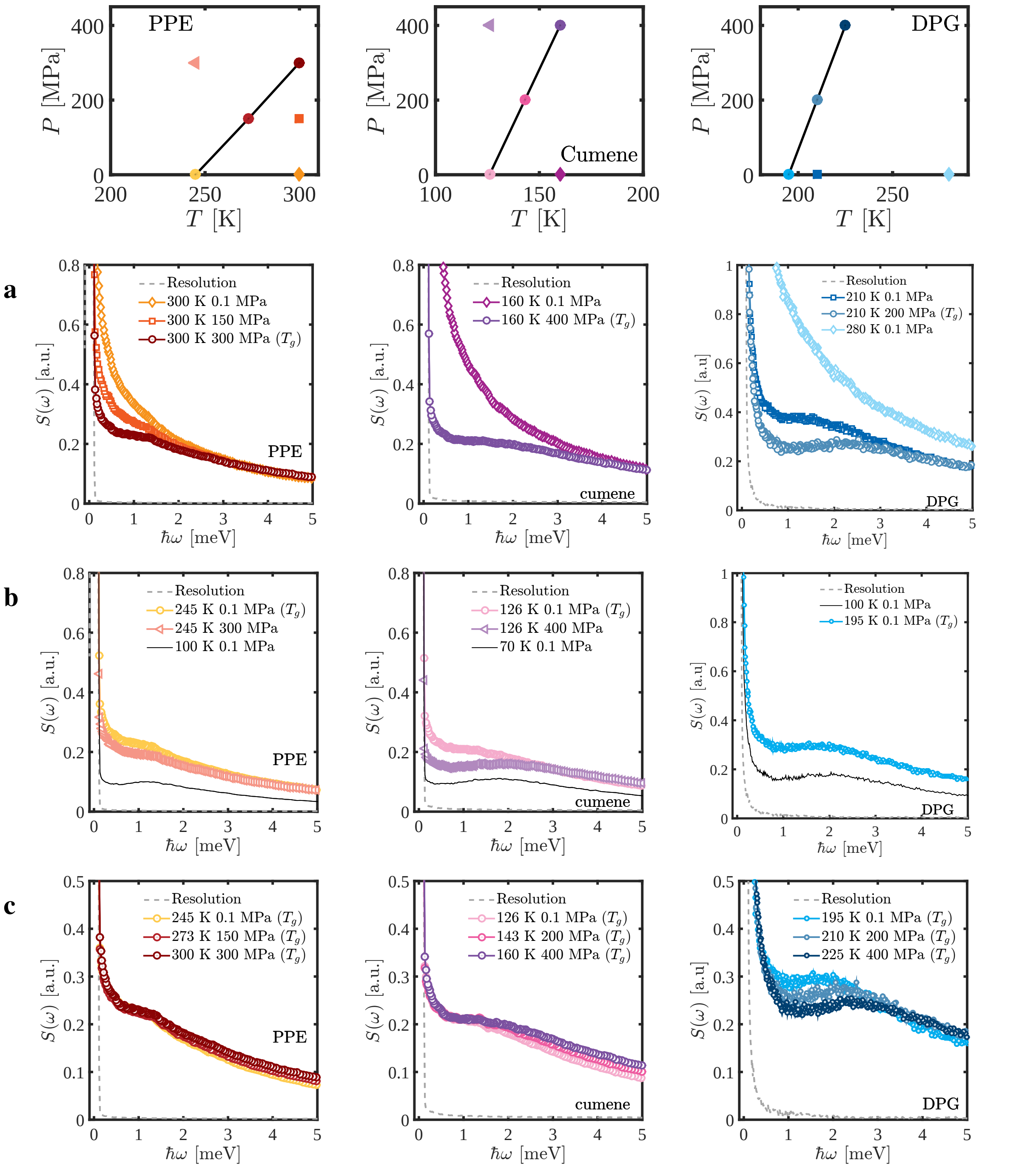

Supplementary Figure 1: Data from Fig. 2 on absolute energy scale. Picosecond dynamics at various state points for the three samples, PPE, cumene and DPG as a function of energy transfer on an absolute energy scale. Same state points and spectra as in Fig. 2, but here shown as measured on absolute energy scale.

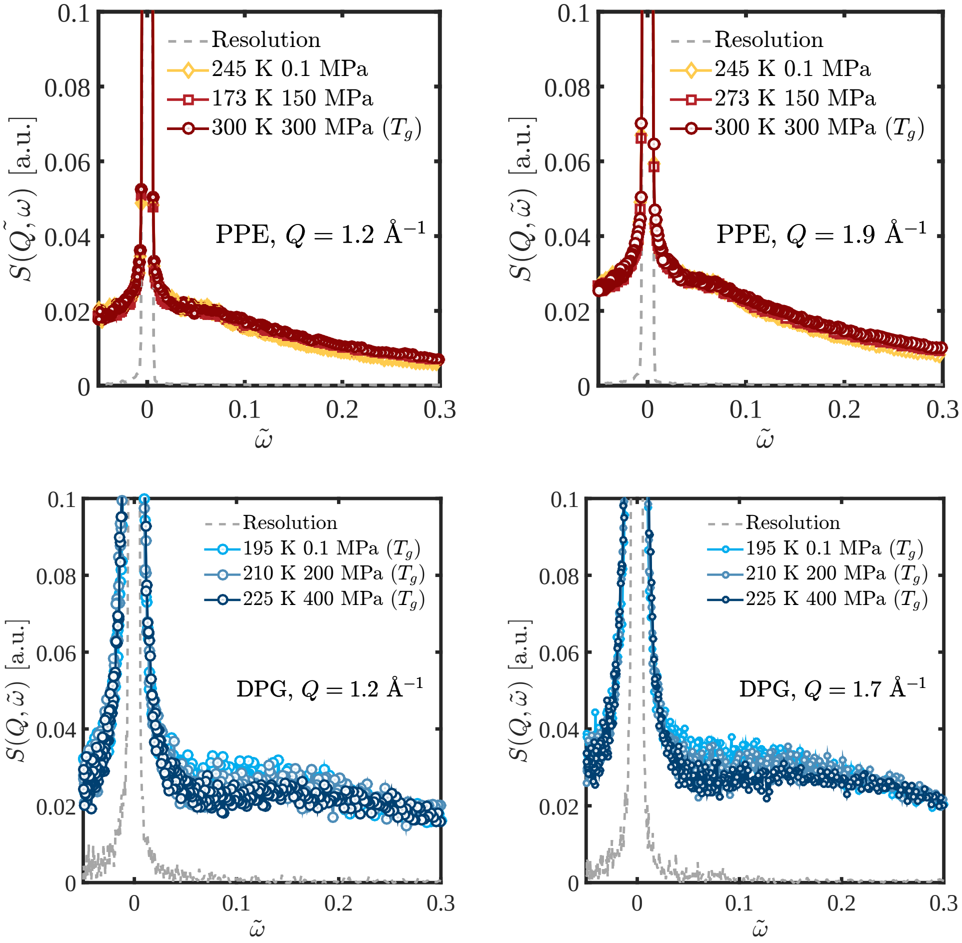

Supplementary Figure 2: Examples of data at individual values of Q. Top: spectra of PPE. Bottom: spectra of DPG. Left: lowest value of Q. Right: higest value of Q. Same trend observed for all values of Q. Spectra plotted in reduced energy units $\tilde{\omega} = \omega\rho^{-1/3}T^{-1/2}$. Spectra in the main paper are summed over Q.
